# Complete Genome Sequence of a *Klebsiella pneumoniae* Strain Carrying Novel Variant *bla*_KPC-203_, Cross-Resistant to Ceftazidime/Avibactam and Cefiderocol, but Susceptible to Carbapenems, Isolated in Italy, 2023

**DOI:** 10.3390/pathogens13060507

**Published:** 2024-06-15

**Authors:** Stefano Amadesi, Gabriele Bianco, Benedetta Secci, Teresa Fasciana, Matteo Boattini, Cristina Costa, Paolo Gaibani

**Affiliations:** 1 Microbiology Unit, IRCCS Azienda Ospedaliero-Universitaria di Bologna, 40126 Bologna, Italy; stefano.amadesi@unibo.it (S.A.); benedetta.secci@unibo.it (B.S.); paolo.gaibani@univr.it (P.G.); 2 Department of Experimental Medicine, University of Salento, 73100 Lecce, Italy; 3 Microbiology and Virology Unit, University Hospital Città della Salute e della Scienza di Torino, 10126 Turin, Italy; matteo.boattini@unito.it (M.B.); cristina.costa@unito.it (C.C.); 4 Department of Health Promotion, Maternal and Child Health, Internal Medicine and Specialty Excellence “G. D’Alessandro”, University of Palermo, 90127 Palermo, Italy; teresa.fasciana@virgilio.it; 5 Department of Public Health and Paediatrics, University of Torino, 10126 Turin, Italy; 6 Lisbon Academic Medical Centre, 1649-028 Lisbon, Portugal; 7 Department of Diagnostic and Public Health, Microbiology Section, Verona University, 37134 Verona, Italy

**Keywords:** *Klebsiella pneumoniae*, KPC-203, carbapenemase detection, KPC variants, ceftazidime/avibactam resistance, cefiderocol resistance, genome sequencing

## Abstract

Background: *Klebsiella pneumoniae* is a concerning pathogen, responsible for hospital-associated outbreaks. Multi drug resistant (MDR) strains are especially hard to treat. We conducted whole-genome sequencing on a MDR *K. pneumoniae* strain in order to identify genomic features potentially linked to its phenotype. Methods: DNA sequencing was performed on the Illumina iSeq 100 platform. Genome assembly was carried out with SPAdes. The genome was annotated with RASTtk. Typing was performed with MLST and Kaptive. Antibiotic resistance genes were detected with AMRFinderPlus and Abricate, and further verified with BLAST. Results: The strain exhibited resistance to ceftazidime/avibactam and cefiderocol, but remained susceptible to carbapenems. The strain belonged to sequence type ST101, serotype O1:K17. The analysis of antibiotic resistance genes indicated that the strain carried a novel KPC variant, designated as KPC-203, featuring a EL deletion at amino acid position 166–167, within the Ω-loop, and a nine-amino-acid insertion (LAVYTRAPM) at position 259. Sequence alterations were found in porin genes *ompK35* and *ompK36*. Unlike molecular testing, which was able to detect the KPC-203 variant, all phenotypic carbapenemase detection methods achieved negative results. Conclusions: KPC-203, a novel KPC variant, showed a sequence modification in a cephalosporin resistance-associated hotspot. Interestingly, such alterations typically correlate with the restoration of carbapenem susceptibility. We hypothesize that KPC-203 likely led to resistance to ceftazidime/avibactam and cefiderocol, while maintaining susceptibility to carbapenems.

## 1. Introduction

*Klebsiella pneumoniae* is a Gram-negative opportunistic pathogen of significant concern due to its capacity to disseminate in clinical settings, resulting in high mortality rates [1]. Carbapenemase-producing strains are of particular significance owing to their ability to hydrolyze main β-lactam antibiotics [2]. Carbapenemase-producing Klebsiella pneumoniae (KPC-Kp) has emerged globally as one of the most clinically relevant multidrug-resistant pathogens due to the limited number of effective therapeutic options, the high mortality rates of associated infections, and its widespread ability to spread in health care facilities worldwide [3]. Among the newly approved drugs, β-lactam/β-lactam inhibitor combinations such as ceftazidime/avibactam have been widely used in the clinic to treat KPC-Kp infections in recent years [4,5]. The use of ceftazidime/avibactam has been associated with a reduction in both mortality rates and the prescription of carbapenems, aminoglycosides, and tigecycline when compared to the other regimens [5]. However, in vivo selection events of ceftazidime/avibactam-resistant strains and nosocomial outbreaks of ceftazidime/avibactam-resistant KPC-Kp have been reported in Italy soon after the drug was introduced into the clinical setting [6,7]. Acquired resistance to ceftazidime/avibactam is mainly due to amino acid substitutions in β-lactamases, alterations in OmpK35/36 porins, and/or the overexpression of efflux pumps. Currently, from an epidemiological point of view, the most common resistance mechanism is the expression of KPC variants characterized by single amino acid substitutions between positions 164–179 in the Ω-loop region [8,9]. These variants are characterized by impaired carbapenemase activity and the restoration of carbapenem susceptibility. Moreover, while molecular testing is capable of detecting all KPC Ω-loop mutants, lateral flow immunoassays and main phenotypic carbapenamse detection methods fail to detect these variants with diminished carbapenemase activity [7,10,11].

In this study, we analyzed the genome of a *K. pneumoniae* clinical strain harboring a novel KPC variant, characterized by impaired carbapenemase activity and potentially implicated in the development of resistance to ceftazidime/avibactam.

## 2. Materials and Methods

### 2.1. Bacterial Strain

The *K. pneumoniae* strain (named 11pa15) was isolated on the 15 October 2022 from a blood sample collected from an 84-year-old male patient hospitalized at G. F. Ingrassia Hospital in Palermo, Italy. Species identification was performed using the MALDI Biotyper (Bruker, Billerica, MA, USA) system. Antimicrobial susceptibility testing was performed using the Sensititre EUMDROXF panel (ThermoFisher Scientific, Monza, Italy) for amikacin, aztreonam, cefepime, ceftazidime/avibactam, ceftolozane/tazobactam, eravacycline, fosfomycin, imipenem, meropenem, piperacillin/tazobactam, tigecyclin, tobramycin, meropenem/vaborbactam, and imipenem/relebactam. Cefiderocol susceptibility was determined by reference broth microdilution using ID-CAMHB (Bruker Daltonics GmbH Co. KG, Bremen, Germany), with a cefiderocol concentration range of 0.03–32 mg/L. MICs were interpreted following the EUCAST guidelines (v13.1).

### 2.2. Carbapenemase Detection

Carbapenemase detection was performed using a commercial genotypic assay (Xpert^®^ Carba-R; Cepheid, Sunnyvale, CA, USA). Five phenotypic carbapenemase detection methods were evaluated: the modified carbapenem inactivation method (mCIM), Rapidec CarbaNP (Biomerieux, Marcy-l’Étoile, France), the Disc Diffusion Synergy test (KPC, MBL and OXA-48 Confirm Kit, Rosco Diagnostica, Albertslund, Denmark), and two LFIAs (NG-Test CARBA 5- NG Biotech, Guipry-Messac, France; RESIST-5 O.O.K.N.V- Coris Bioconcept, Gembloux, Belgium).

### 2.3. Whole-Genome Sequencing and Bioinformatics

Whole-genome DNA sequencing was executed in order to characterize the genomic features associated with the strain phenotype. Total DNA was extracted from overnight culture on Mueller–Hinton agar plates (ThermoFisher Scientific, Monza, Italy) using the Dneasy Blood & Tissue Kit (Qiagen, Hilden, Germany) and cleaned up using the AMPure XP Bead-Based Reagent (Beckman Coulter, Brea, CA, USA). Paired-end libraries were prepared using the Illumina DNA Prep Kit and sequenced on the Illumina iSeq 100 system (Illumina, San Diego, CA, USA). Read quality was evaluated with FastQC v0.12.1 (https://www.bioinformatics.babraham.ac.uk/projects/fastqc; accessed on 1 January 2024), followed by adapter trimming with Trimmomatic v0.39 (https://github.com/usadellab/Trimmomatic; accessed on 1 January 2024). Genome assembly was carried out using SPAdes v3.15.2 (https://github.com/ablab/spades; accessed on 1 January 2024). Multi-locus sequence type was determined by comparing the genome with schemes from the PubMLST website (https://pubmlst.org/; accessed on 1 January 2024) using MLST v2.23.0 (https://github.com/tseemann/mlst; accessed on 1 January 2024), while serotype identification was performed with Kaptive v2.0.6 (https://github.com/klebgenomics/Kaptive; accessed on 1 January 2024). Plasmid replicon types were determined with PlasmidFinder v2.1.6 (https://cge.food.dtu.dk/services/PlasmidFinder/; accessed on 1 January 2024). Genomic features were predicted using RASTtk v4.0. (https://github.com/TheSEED/RASTtk-Distribution; accessed on 1 January 2024). Genes correlated with antibiotic resistance, virulence and stress response were detected with AMRFinderPlus v3.11.14 (https://github.com/ncbi/amr; accessed on 1 January 2024) and Abricate v1.0.1 (https://github.com/tseemann/abricate), and the sequences were further investigated using BLAST v2.14.0 (https://blast.ncbi.nlm.nih.gov/Blast.cgi; accessed on 1 January 2024).

## 3. Results

The antimicrobial susceptibility pattern of the *K. pneumoniae* 11pa15 strain is shown in Table 1. The strain showed resistance to ceftazidime/avibactam and cefiderocol (MICs > 16 μg/mL and 4 μg/mL, respectively), susceptibility to meropenem, imipenem (MICs 1 μg/mL and ≤1 μg/mL, respectively), and to combinations of meropenem/vaborbactam and imipenem/relebactam (MICs 0.5 μg/mL and 0.25 μg/mL, respectively).

A total of 1,163,626 paired-end reads with a length of 151 bp were obtained by Illumina sequencing. Genome assembly produced a draft with a total size of 5,671,248 bp, composed of 72 contigs ranging from 505,895 to 282 bp in length (median length 27,477 bp). The genome had a 56.84% G+C content, an N50 of 223,646, and 30× mean coverage. Genome-based typing revealed that the strain belonged to the multi-locus sequence type ST101, serotype O1:K17. In addition, the strain harbored plasmid replicons belonging to the incompatibility types Col156, Col440II, ColRNAI, IncFIA(HI1), IncFIB(Mar), IncFII(K), and IncR. Genes linked with resistance to several antibiotics were found, including aminoglycosides (*aadA1*, *acrD*, *ant(2″)-Ia*, *armA*, *cpxA*, *cpxR*), β-lactams (*ampH*, *bla*_KPC_, *bla*_SHV-1_), chloramphenicol (*catA1*), fosfomycin (*fosA*), macrolides (*mph(E)*, *msr(E)*), quinolones (*oqxA*, *oqxB20*, *gyrA*), sulfonamides (*sul1*), tetracyclines (*rpsJ*), as well as multiple genes involved in multi-drug efflux (*acrA*, *acrB*, *asmA*, *baeR*, *baeS*, *crp*, *emrD*, *emrR*, *kpnE-H*, *marA*, *mdfA*, *mdtA-C*, *msbA*, *phoP*, *phoQ*, *pmrA*, *pmrB*, *pmrD*, *ramA*, *smvA*, *soxS*). The sequence analysis of the porin genes associated with carbapenem resistance revealed that *ompK35* had a sequence interruption at amino acid position 62, while *ompK36* exhibited a double insertion (aspartic acid and threonine) at amino acid position 136. No alterations were found in *ompK37*. Virulence genes with roles in invasion (*ompA*), pilus formation (*yagV-Z*, *ykgK*), yersiniabactin (*fyuA*, *irp1*, *irp2*, *ybtA*, *ybtE*, *ybtP*, *ybtQ*, *ybtS*, *ybtT*, *ybtU*, *ybtX*) and enterobactin (*entA*, *entB*, *fepC*) biosynthesis were detected. Sequence analysis of the *bla*_KPC_ gene revealed that the strain carried KPC-203, a KPC-3 variant never described before. This variant had a 96.36% sequence identity to KPC-3 and exhibited a double deletion (glutamic acid and leucine) at amino acid positions 166 and 167, as well as a nine-amino-acid long (LAVYTRAPM) insertion at position 259. Unlike Xpert^®^ Carba-R, molecular testing, which was able to detect the KPC-203 variant, all phenotypic carbapenemase detection methods achieved negative results. 

## 4. Discussion

The development of new KPC variants associated with ceftazidime/avibactam resistance is a public alarm: to date, more than 150 *bla*_KPC_ variants have been reported worldwide, and most of the new variants have been discovered in the past 3 years [12,13]. Many of the discovered variants are derived from the wild-type forms KPC-2 and KPC-3 and have mutations in the omega loop region, among the most reported being KPC-31 and KPC-33. Currently, there are still no guidelines or expert consensus to make recommendations for the diagnosis and treatment of infections caused by bacteria producing the new KPC variants. 

Herein, we characterized the genome of a ceftazidime/avibactam-resistant *K. pneumoniae* clinical strain carrying a novel KPC-3 variant, designated as KPC-203. This variant exhibited a double deletion within the Ω-loop (residues 164–179), a well-established hotspot associated with CAZ/AVI-resistance. Mutations in the omega loop domain result in an increased affinity for ceftazidime and a weakened affinity for avibactam by changing the structure of KPC, thus mediating bacterial resistance to ceftazidime/avibactam [12,13]. Moreover, the strain exhibited a low-level resistance to cefiderocol, a new approved drug approved for treatment of multi drug resistant Gram-negative infections. This finding is in agreement with several reports that showed that specific mutations in the omega loop of the KPC enzyme can lead to increased hydrolysis of cefiderocol, which is structurally similar to ceftazidime, resulting in cross-resistance toward both ceftazidime/avibactam and cefiderocol [14,15,16,17]. 

Notably, the deletion within the Ω-loop (residues 164–179) observed in the *K. pneumoniae* 11pa15 strain was associated with carbapenems susceptibility and the negativity of the main phenotypic carbapenemase detection methods, including both commercial lateral flow immunossays. This represents an important diagnostic issue, as the rapid identification of KPC is essential in treating carbapenemase-producing Enterobacterales infections [5]. The emergence of KPC variants upsets the conventional thinking of clinical microbiologists about carbapenemase detection and discourages the practice of inferring susceptibility to ceftazidime/avibactam from carbapenemase detection results. Moreover, since phenotypic assays have been highly favored due to their low cost, they are therefore widely used for the confirmation of carbapenemase production in active surveillance programs. Obtaining false-negative results for carbapenemases causes strains expressing these new variants to escape active surveillance programs, facilitating easy dissemination in the hospital setting [7,18]. Based on the above information, the spread of KPC variants requires the implementation of measures, such as: i. performing ceftazidime/avibactam susceptibility testing in conjunction with carbapenemase testing; ii. performing molecular testing for carbapenemase detection or the application of diagnostic algorithms based on both genotypic and phenotypic testing [5]; iii. implementing selective and differential culture media in active surveillance programs to highlight the ceftazidime/avibactam-resistant isolates [18].

The study has some limitations. The main limitation is that we assessed the KPC-203 enzyme variant using genomic sequence analysis and phenotype expressed by the *K. pneumoniae* strain. Further studies are warranted to functionally characterize KPC-203 in order to confirm the direct association between the new KPC variant and the observed resistance phenotype, thus excluding the impact of other resistance mechanisms. Secondly, the medical history of the patient from whom the bacterial strain was isolated, including the source of infection and the antimicrobial therapy that led to the selection of the strain, was not investigated.

## 5. Conclusions

Here, we characterized the genome of a *K. pneumoniae* clinical isolate carrying a novel KPC-3 variant, designated as KPC-203, and associated with cross-resistance to ceftazidime/avibactam and cefiderocol. Notably, modifications in this region have been documented to potentially restore susceptibility to carbapenems, which may explain the phenotypic profile of our strain. In conclusion, future research should focus on reporting novel KPC variants, given their relevance in the context of clinical outbreaks. In addition, the development of rapid tests able to identify KPC variants associated with resistance to new β-lactam/β-lactamase inhibitor combinations is desirable.

## Figures and Tables

**Table 1:** MIC values for all compounds tested on the *K. pneumoniae* 11pa15 strain and respective interpretation according to the latest EUCAST guidelines (v13.1) (https://www.eucast.org/clinical_breakpoints accessed on 1 January 2024).

Antibiotic	MIC (μg/mL)	MIC Interpretation
amikacin	>32	resistant
aztreonam	≤1	susceptible
cefepime	16	resistant
ceftazidime/avibactam	>16	resistant
ceftolozane/tazobactam	>8	resistant
eravacycline	>0.5	resistant
fosfomycin	>64	resistant
imipenem	≤1	susceptible
meropenem	1	susceptible
piperacillin/tazobactam	8	susceptible
tigecyclin	≤0.5	susceptible
tobramycin	>4	resistant
meropenem/vaborbactam	0.5	susceptible
imipenem/relebactam	0.25	susceptible
cefiderocol	4	resistant

## Data Availability

The draft genome assembly of the strain 11pa15 has been deposited in the NCBI BioSample database under accession number SAMN38513653. The amino acid sequence of KPC-203 can be found in the Protein database under accession number MDY6716274.1.

## References

[1] Wang G., Zhao G., Chao X., Xie L., Wang H. (2020). The Characteristic of Virulence, Biofilm and Antibiotic Resistance of *Klebsiella pneumoniae*. Int. J. Environ. Res. Public Health.

[2] Chen L., Mathema B., Chavda K.D., DeLeo F.R., Bonomo R.A., Kreiswirth B.N. (2014). Carbapenemase-producing *Klebsiella pneumoniae*: Molecular and genetic decoding. Trends Microbiol..

[3] Tzouvelekis L.S., Markogiannakis A., Psichogiou M., Tassios P.T., Daikos G.L. (2012). Carbapenemases in Klebsiella pneumoniae and other Enterobacteriaceae: An evolving crisis of global dimensions. Clin. Microbiol. Rev..

[4] Tumbarello M., Raffaelli F., Giannella M., Mantengoli E., Mularoni A., Venditti M., De Rosa F.G., Sarmati L., Bassetti M., Brindicci G. (2021). Ceftazidime-Avibactam Use for *Klebsiella pneumoniae* Carbapenemase-Producing *K. pneumoniae* Infections: A Retrospective Observational Multicenter Study. Clin. Infect. Dis..

[5] Boattini M., Bianco G., Charrier L., Comini S., Iannaccone M., Almeida A., Cavallo R., De Rosa F.G., Costa C. (2023). Rapid diagnostics and ceftazidime/avibactam for KPC-producing Klebsiella pneumoniae bloodstream infections: Impact on mortality and role of combination therapy. Eur. J. Clin. Microbiol. Infect. Dis..

[6] Gaibani P., Bovo F., Bussini L., Bartoletti M., Lazzarotto T., Viale P., Pea F., Ambretti S. (2023). Colonization by ceftazidime/avibactam-resistant KPC-producing Klebsiella pneumoniae following therapy in critically ill patients. Clin. Microbiol. Infect..

[7] Bianco G., Boattini M., Bondi A., Comini S., Zaccaria T., Cavallo R., Costa C. (2022). Outbreak of ceftazidime-avibactam resistant *Klebsiella pneumoniae* carbapenemase (KPC)-producing *Klebsiella pneumoniae* in a COVID-19 intensive care unit, Italy: Urgent need for updated diagnostic protocols of surveillance cultures. J. Hosp. Infect..

[8] Gaibani P., Giani T., Bovo F., Lombardo D., Amadesi S., Lazzarotto T., Coppi M., Rossolini G.M., Ambretti S. (2022). Resistance to Ceftazidime/Avibactam, Meropenem/Vaborbactam and Imipenem/Relebactam in Gram-Negative MDR Bacilli: Molecular Mechanisms and Susceptibility Testing. Antibiotics.

[9] Carattoli A., Arcari G., Bibbolino G., Sacco F., Tomolillo D., Di Lella F.M., Trancassini M., Faino L., Venditti M., Antonelli G. (2021). Evolutionary Trajectories toward Ceftazidime-Avibactam Resistance in *Klebsiella pneumoniae* Clinical Isolates. Antimicrob. Agents Chemother..

[10] Zhang J., Xu J., Shen S., Ding L., Yang W., Tang C., Shi Q., Zhao H., Guo Y., Han R. (2024). Comparison of three colloidal gold immunoassays and GeneXpert Carba-R for the detection of *Klebsiella pneumoniae bla*_KPC-2_ variants. J. Clin. Microbiol..

[11] Hernández-García M., Castillo-Polo J.A., Cordero D.G., Pérez-Viso B., García-Castillo M., Saez de la Fuente J., Morosini M.I., Cantón R., Ruiz-Garbajosa P. (2022). Impact of Ceftazidime-Avibactam Treatment in the Emergence of Novel KPC Variants in the ST307-Klebsiella pneumoniae High-Risk Clone and Consequences for Their Routine Detection. J. Clin. Microbiol..

[12] Hobson C.A., Pierrat G., Tenaillon O., Bonacorsi S., Bercot B., Jaouen E., Jacquier H., Birgy A. (2022). *Klebsiella pneumoniae* Carbapenemase Variants Resistant to Ceftazidime-Avibactam: An Evolutionary Overview. Antimicrob. Agents Chemother..

[13] Ding L., Shen S., Chen J., Tian Z., Shi Q., Han R., Guo Y., Hu F. (2023). *Klebsiella pneumoniae* carbapenemase variants: The new threat to global public health. Clin. Microbiol. Rev..

[14] Bianco G., Boattini M., Comini S., Iannaccone M., Bondi A., Cavallo R., Costa C. (2022). In vitro activity of cefiderocol against ceftazidime-avibactam susceptible and resistant KPC-producing Enterobacterales: Cross-resistance and synergistic effects. Eur. J. Clin. Microbiol. Infect. Dis..

[15] Hobson C.A., Cointe A., Jacquier H., Choudhury A., Magnan M., Courroux C., Tenaillon O., Bonacorsi S., Birgy A. (2021). Cross-resistance to cefiderocol and ceftazidime-avibactam in KPC β-lactamase mutants and the inoculum effect. Clin. Microbiol. Infect..

[16] Birgy A., Nnabuife C., Palzkill T. (2024). The mechanism of ceftazidime and cefiderocol hydrolysis by D179Y variants of KPC carbapenemases is similar and involves the formation of a long-lived covalent intermediate. Antimicrob. Agents Chemother..

[17] Poirel L., Sadek M., Kusaksizoglu A., Nordmann P. (2022). Co-resistance to ceftazidime-avibactam and cefiderocol in clinical isolates producing KPC variants. Eur. J. Clin. Microbiol. Infect. Dis..

[18] Bianco G., Boattini M., Comini S., Leone A., Bondi A., Zaccaria T., Cavallo R., Costa C. (2022). Implementation of Chromatic Super CAZ/AV^I®^ medium for active surveillance of ceftazidime-avibactam resistance: Preventing the loop from becoming a spiral. Eur. J. Clin. Microbiol. Infect. Dis..

